# Glial heterogeneity in the primate spinal cord: Implications of age and sex differences for neurodegenerative and neurotraumatic diseases

**DOI:** 10.4103/NRR.NRR-D-25-01274

**Published:** 2026-01-27

**Authors:** Chloé M. Gazard, Gaëtan Poulen, Florence E. Perrin

**Affiliations:** MMDN, Univ. Montpellier, EPHE, INSERM, Montpellier, France; Department of Neurosurgery, CHU, Montpellier, France; Universitaire de France (IUF), Paris, France

The central nervous system (CNS) relies on the intricate interplay between neurons and glial cells to maintain homeostasis and coordinate responses to disease or injury. It is now well established that within this network, astrocytes, microglia, and oligodendrocytes are critical regulator of neural function, actively shaping metabolism, inflammation, and therefore degenerative and repair processes. Glial dysfunction and alteration of dynamic cross-communication among the glial cell population have emerged as a common denominator in both neurodegenerative and neurotraumatic conditions. It is therefore clear that glial cells are not passive bystanders but active contributors to CNS health and disease.

Despite growing interest in glial biology, significant gaps remain. Most research has focused on the brain, leaving the spinal cord relatively understudied. Yet the spinal cord displays a unique architecture and is particularly vulnerable to trauma and neurodegenerative conditions that often result in lifelong disability. Most preclinical analyses rely on rodent models, which have provided invaluable insights but only partially recapitulate the complexity of human glial biology. Importantly, endogenous variability in glial cells driven by factors such as regional specialization, sex, and age can significantly influence the functional states of glial cells and therefore influences disease susceptibility, disease onset, and progression, and therapeutic responsiveness. Addressing these dimensions is critical to bridging translational gaps and developing more personalized interventions.

In this objective, our recent study investigated age- and sex-dependent variations in glial cells and myelin within the healthy spinal cords of both *Microcebus murinus* (a small nonhuman primate [NHP]) and humans (Poulen et al., 2025). Using immunohistochemistry and myelin staining techniques, we analyzed spinal cord samples from midlife and aged individuals of both sexes and species. We observed a sex-dependent expression of the astrocytic marker glial fibrillary acidic protein in both species, suggesting that astrocytic responses may be modulated by sex-specific factors, potentially influencing susceptibility to spinal cord pathologies. We also identified pronounced differences in microglial marker expression, including ionized calcium-binding adaptor molecule 1 and breast cancer 1 (Noristani et al., 2015). These differences were evident across age groups and between sexes, indicating that microglial activity and function are highly sensitive to both aging and sex-related factors.

Moreover, we found species-specific patterns in the expression of the oligodendrocytic marker 2′,3′-cyclic nucleotide 3′-phosphodiesterase and in myelin parameters. The g-ratio, which reflects the efficiency of nerve conduction, displayed opposite trends in *Microcebus murinus* compared to humans. Deviations from the normal g-ratio can indicate myelin thinning or loss, potentially contributing to disorders like multiple sclerosis or myelin degeneration following traumatic injury. This highlights the importance of species-specific evidence when investigating myelin dynamics. Finally, in human samples, we identified an age-related decrease in myelin thickness, particularly in women. This decline correlated with an increased g-ratio, suggesting a potential mechanism underlying age-associated spinal cord vulnerability and emphasizing the need to also consider sex differences in aging evidence.

Variations in glial marker expression and myelin structure in the healthy primate spinal cord highlight the potential role of glial cells in driving age- and sex-related differences in the prevalence and progression of spinal cord diseases. Crucially, these differences may also drive distinct therapeutic responses in men and women.

**Glial heterogeneity and endogenous variability: *Regional specialization*:** Glial properties vary across the CNS. The spinal cord, in particular, displays a distinct glial composition and organization compared to the brain (for review, see Yoon et al., 2017; Xuan et al., 2019). Astrocytic domains are more tightly organized, microglia exhibit unique surveillance dynamics, and oligodendrocytes are distributed according to tract-specific requirements for conduction speed. This regional specialization indicates that findings from cortical or hippocampal glia cannot be extrapolated uncritically to the spinal cord, especially in the context of trauma or degeneration (**Figure 1**).

**Figure 1:**
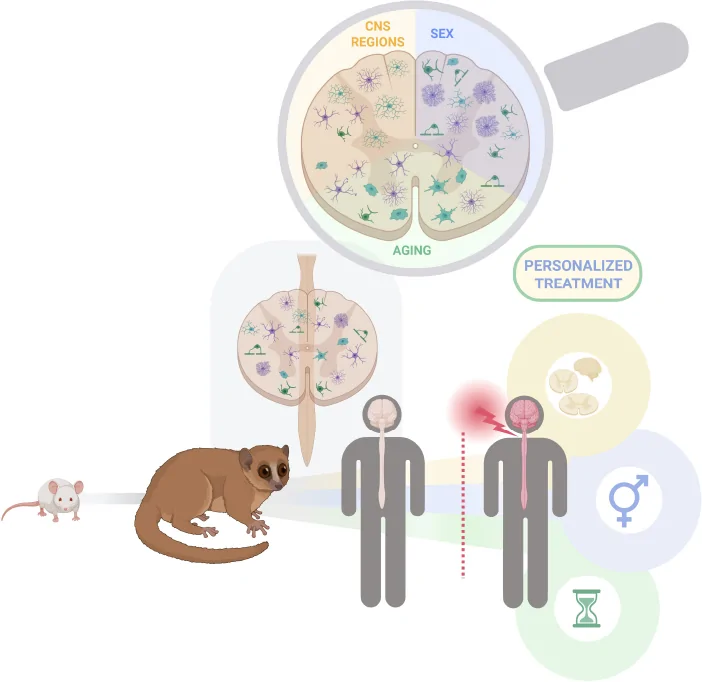
Regional specialization, sex, and age influences on glia: Insights from nonhuman primates for human translation. Glial phenotypes in the central nervous system (CNS) are modulated by region, sex, and age. Nonhuman primates represent a robust translational model to capture this heterogeneity and provide a relevant framework to inform the development of personalized therapeutic strategies in CNS diseases and injuries. Created in BioRender (https://biorender.com/2u22ur2).

***Sex-dependent variability:*** Sex differences also profoundly shape glial biology. Hormonal regulation affects astrocytic reactivity, microglial immune responses, and oligodendrocyte maturation (for review see O’Connor and Nissen, 2023; Bortolanza et al., 2025). For instance, oestrogens can promote remyelination and drive microglia toward anti-inflammatory phenotypes, whereas androgens may influence astrocytic proliferation and glial scar formation. Beyond hormones, sex-specific genetic and epigenetic mechanisms further contribute to divergent glial phenotypes (**Figure 1**).

***Age-related changes:*** Aging is accompanied by profound modifications in glial biology. Microglia in aged individuals often adopt a “primed” state, characterized by reduced tissue surveillance and impaired resolution of inflammation (for review, see Edler et al., 2021). This contributes to chronic neuroinflammation, diminished plasticity, and increased vulnerability to both neurodegenerative and neurotraumatic insults. Astrocytes also undergo senescence-like changes: they shrink in size, exhibit reduced ramification and complexity, lose their homeostatic and neuroprotective functions, and acquire detrimental properties (for review, see Verkhratsky and Semyanov, 2023). Oligodendrocytes, crucial for myelin production and maintenance, show reduced numbers and a diminished capacity to proliferate and remyelinate, further exacerbating demyelination and limiting repair after injury (**Figure 1**).

**Epidemiology of age- and sex-dependent neurodegenerative and neurotraumatic disorders:** Sex and age significantly influence the prevalence and clinical course of neurological diseases, including those affecting the spinal cord, through direct effects such as hormones, genetics, and immune responses, as well as indirect factors including epigenetics, lifestyle, nutrition, and gut microbiota (for review see Bianco et al., 2023; Perrin et al., 2025). In multiple sclerosis, the female-to-male ratio reaches 3:1, with onset typically between 20 and 40 years. Alzheimer’s disease, primarily affecting individuals over 65 years (rare early-onset cases at 30–60 years), shows a female predominance (2:1), while Parkinson’s disease is more common in males (1:1.5), usually after age 60. Amyotrophic lateral sclerosis exhibits a modest male predominance (1:1.2–1.5), peaking between 55–75 years, whereas spinal muscular atrophy displays variable onset from infancy to adulthood depending on subtype.

In neurotraumatic disorders, lifestyle factors strongly shape sex ratios. Traumatic brain injury is nearly twice as common in males. For traumatic spinal cord injury (TSCI), adult males are at least twice as likely to sustain an injury compared to females, with peaks in young adulthood (15–19 years in females; 20–29 years in males) and later life (≥ 60 years in women; ≥ 70 years in men). Interestingly, pediatric TSCI shows a more balanced sex distribution. Over recent decades, the mean age at TSCI onset has increased substantially; in the United States, it rose from 29 years in the 1970s to over 43 years after 2015, reflecting demographic shifts (for review, see Perrin et al., 2025).

Together, these epidemiological patterns highlight how sex- and age-dependent differences in glial biology may critically shape disease risk, progression, and therapeutic outcomes.

**Glial responses in neurodegenerative and neurotraumatic disorders:** Glial cells are key players in both neurodegenerative and neurotraumatic conditions, with roles that vary according to disease context and CNS region. Importantly, their involvement extends beyond classical disorders to include conditions such as neuropathic pain and CNS tumors.

***Neurodegeneration — chronic and progressive dysfunction:*** Neurodegenerative diseases typically have an insidious onset and gradual progression, with glial dysfunction accumulating over years, often preceding clinical symptoms. In Alzheimer’s disease and Parkinson’s disease, microglia and astrocytes adopt reactive states that drive chronic neuroinflammation, synaptic dysfunction, and neuronal loss, while oligodendrocyte impairment reduces myelination and metabolic support. In multiple sclerosis, demyelination is largely immune-mediated, with oligodendrocyte loss and persistent microglial and astrocytic roles in sustaining inflammation and metabolic stress. In amyotrophic lateral sclerosis, astrocytes promote motor neuron toxicity, whereas microglia shift from early protective to late-stage neurotoxic roles.

***Neurotrauma — acute and spatially organized responses:*** In contrast, traumatic injuries such as traumatic brain injury and TSCI trigger abrupt and spatially constrained glial responses. Within minutes, microglia and astrocytes activate to contain tissue damage, initiate inflammation, and recruit peripheral immune cells. Astrocytes form borders that restrict lesion expansion but block axonal regrowth. Microglia phagocytose debris but simultaneously release pro-inflammatory mediators that amplify secondary injury. Oligodendrocytes, highly vulnerable to excitotoxicity and oxidative stress, undergo acute loss, leading to rapid demyelination.

***Shared duality:*** Despite these differences, a common principle emerges: glial responses can be protective or detrimental depending on timing, severity, and the individual’s endogenous state (e.g., age, sex, and prior exposures).

***Nonhuman primates — bridging the translational gap:*** Rodents have been instrumental in advancing glial biology, but their limitations are increasingly evident. Rodent glia differ substantially from human glia in size, density, transcriptional profiles, and responses to stimuli. NHPs, in contrast, more faithfully replicate human glial and myelin features. They also allow the study of complex variables such as hormonal cycles, lifespan-associated changes, and sex-specific factors.

Therefore, NHP analyses are particularly valuable for testing therapeutic strategies before clinical translation, ensuring that interventions account for age- and sex-dependent glial variability.

**Conclusion and perspectives:** Glial cells are central to CNS health and disease, shaping vulnerability, progression, and recovery in both neurodegenerative and neurotraumatic conditions. Yet, the influence of regional specialization, sex, and age on glial phenotypes remains insufficiently explored, limiting our understanding of disease mechanisms and therapeutic potential (**Figure 1**). Overcoming current biases, favoring the brain over the spinal cord and rodents over NHPs, will be essential to capture the full spectrum of glial heterogeneity. By integrating species, regional context, sex, and age into future investigations, the field can move toward a more translational view of CNS pathology, positioning glial cells as active determinants of outcomes and as targets for personalized therapies.

*We thank Yannick N. Gerber (Univ. Montpellier, France) for helpful comments on the manuscript.*

## References

[R1] Bianco A, Antonacci Y, Liguori M (2023). Sex and gender differences in neurodegenerative diseases: challenges for therapeutic opportunities. Int J Mol Sci.

[R2] Bortolanza M, Gobbo D, Fang L, Scheller A, Bai X, Kirchhoff F (2025). Sex-specific properties of astrocytes: from development to evolutionary insights. Neurochem Res.

[R3] Edler MK, Mhatre-Winters I, Richardson JR (2021). Microglia in aging and Alzheimer’s disease: a comparative species review. Cells.

[R4] Noristani HN, Sabourin JC, Gerber YN, Teigell M, Sommacal A, Vivanco M, Weber M, Perrin FE (2015). Brca1 is expressed in human microglia and is dysregulated in human and animal model of ALS. Mol Neurodegener.

[R5] O’Connor JL, Nissen JC (2023). The pathological activation of microglia is modulated by sexually dimorphic pathways. Int J Mol Sci.

[R6] Perrin FE, Haynes W, Gerber YN, Lonjon N, Barrey CY (2025). Spinal cord injury and regeneration. Spine surgery.

[R7] Poulen G, Douich N, Gazard CM, Mestre-Frances N, Cardoso M, Bauchet L, Vachiery-Lahaye F, Lonjon N, Gerber YN, Perrin FE (2025). Sex and age differences in glia and myelin in nonhuman primate and human spinal cords: implications for pathology. Cell Death Discov.

[R8] Verkhratsky A, Semyanov A (2023). Astrocytes in ageing. Subcell Biochem.

[R9] Xuan FL, Chithanathan K, Lillevali K, Yuan X, Tian L (2019). Differences of microglia in the brain and the spinal cord. Front Cell Neurosci.

[R10] Yoon H, Walters G, Paulsen AR, Scarisbrick IA (2017). Astrocyte heterogeneity across the brain and spinal cord occurs developmentally, in adulthood and in response to demyelination. PLoS One.

